# A chromosome-level genome assembly of *Sarcophaga princeps* Wiedemann, 1830 (Diptera: Sarcophagidae)

**DOI:** 10.1038/s41597-025-05785-0

**Published:** 2025-08-15

**Authors:** Liangliang Li, Yinghui Wang, Yingna Zhang, Wenxuan Huang, Shujin Li, Yu Wang

**Affiliations:** 1 Shandong University of Political Science and Law, Jinan, 250014 Shandong China; 2 Hebei Key Laboratory of Forensic Medicine, Shijiazhuang, 050017 Hebei China; 3 Department of Forensic Medicine, Soochow University, Suzhou, 215000 Jiangsu China; 4 Department of Anatomy, Shihezi University School of Medicine, Shihezi, 832003 Xinjiang China

**Keywords:** Entomology, Genome

## Abstract

Sarcosaprophagous insects play a pivotal role in forensic entomology, particularly in estimation of the postmortem interval (PMI). Among them, *Sarcophaga princeps* Wiedemann, 1830 (Diptera: Sarcophagidae), demonstrates distinct ecological traits such as larviposition and the ability to colonize buried remains. Even so, it has been hard to learn more about its molecular biology since a high-quality genome assembly is missing. This research uses PacBio HiFi, Illumina and Hi-C methods to produce a chromosome-level genome assembly for *S. princeps*. The resulting genome spans 628.56 Mb, with a scaffold N50 of 118.89 Mb, and 99.87% of the assembled sequence (627.74 Mb) successfully anchored to seven chromosomes. BUSCO analysis indicates that the genome is 99.10% complete, with 94.4% of genes present in single copy and 4.7% duplicated. Annotation of the genome identified 14,348 protein-coding genes and found that repetitive sequences occupy 333.04 Mb of the genome. The availability of this high-resolution genomic resource will greatly improve future research on the evolution, habitats and genomics of the Sarcophagidae family.

## Background & Summary

In forensic entomology, sarcosaprophagous insects are usually chosen to solve technical problems related to death by using their successional and developmental patterns, among which the accurate estimation of the postmortem interval (PMI) is the most important research content and one of the main tasks of forensic investigation^1,2^. After hundreds of millions of years, sarcosaprophagous insects have evolved a way to fit into the ecosystem of corpses that are decaying. Some insects like fresh tissues full of fluids, others like tissues that are decayed or dried out and some obtain their nutrition from other insects^3^. The pattern of succession, in which groups of insects visit corpses at different stages of decomposition in a particular order, lay their offspring and then leave the body in a predictable sequence, can be used in forensic investigations^4,5^.

Sarcophagidae are an important group of sarcosaprophagous insects, usually colonizing the corpses almost simultaneously with Calliphoridae^1,4^. However, due to limitations in taxonomic and biological data, Sarcophagidae have been less well studied and applied. The fact that most Sarcophagidae lay larvae rather than eggs and because their larvae are larger than those of Calliphoridae larvae of the same developmental stage, means that they may be found more easily at the scene of the crime. There are even cases where the Sarcophagidae may be the most important insect evidence at the crime scene^6,7^.

*Sarcophaga princeps* Wiedemann, 1830, is a sarcosaprophagous fly that is widespread in the Eastern Oceanic and Palearctic zones. This species is recognized as ecologically significant in both forensic and medical contexts, as it exhibits the capacity to colonize outdoor human carcasses—serving as an indicator insect for PMI estimation^8,9^—and has been documented to cause hoof fly disease in goats, buffalo, and bulls^10^. In terms of its ecological traits, *S. princeps* is commonly found in association with dead fish, animal carcasses, and other decaying organic matter. It also feeds on flowers of the Aristolochiaceae family, human feces, and dead snails^11^, thereby playing a pivotal role in ecological material cycling.

Its key biological characteristics are summarized as follows: First, the larvae possess highly efficient carrion-decomposing capabilities, which may be linked to the expansion of decomposition-related gene families within their genome. The intestinal microbiota facilitates substance decomposition and pathogen resistance, while the intestinal structure enhances nutrient absorption and immune defense. Additionally, the metabolic system of *S. princeps* can degrade toxins present in carrion, enabling it to occupy a distinct ecological niche. Second, its reproductive strategy reflects adaptive evolutionary selection. Adult individuals exhibit larviparous behavior; this ovoviviparous mode enhances larval survival rates and enables rapid access to food sources, and a trait potentially associated with specific genetic features regulating embryonic development. Finally, this species can colonize both buried and indoor cadavers, a capability that may be related to its olfactory gene repertoire. However, current research on *S. princeps* has primarily focused on molecular identification and systematic classification within the Sarcophagidae^12,13^. The lack of genomic-level data significantly hinders collaborative studies investigating the interactions between this species, cadavers, and the surrounding environment.

To better study the evolution, ecology and colonization of *S. princeps*, we assemble its genome, using PacBio HiFi, Illumina and Hi-C data. Repetitive sequences, non-coding RNAs and protein-coding genes have been annotated by us. High-quality genomes of *S. princeps* play a key role in expanding our knowledge of the Sarcophagidae family, showing important details about their biological habits and ecological characteristics.

## Methods

### Sample collection and sequencing

On April 20, 2023, a male specimen of *S. princeps* was collected in Suzhou, China, to be used for DNA and RNA sequencing. We washed the sample with phosphate-buffered saline for 10 minutes to minimize the risk of outside contamination. The sample was frozen in liquid nitrogen for 20 minutes, after the analysis, and put into storage at −80 °C until sequencing was done in the laboratory.

Genomic DNA and total RNA were extracted using the FastPure® Blood/Cell/Tissue/Bacteria DNA Isolation Mini Kit (Vazyme Biotechnology Co., Ltd., Nanjing, China) and TRIzol reagent (YiFeiXue Technology, Nanjing, China), respectively. PCR-free short-read libraries for whole genome sequencing (WGS) were employing the TruSeq DNA PCR-Free Library Preparation Kit. The libraries were made so that reads would be 150 bp long on both ends, with an average insert size of 350 bp. The Hi-C sequencing library was constructed according to the standard procedure described by Belton *et al*.^14^ with minor modifications. In brief, specimen was ground into pieces and incubated in 2% formaldehyde solution to complete cross-linking. Nuclei were isolated and digested with MboI, then labeled with biotin-14-dCTP. The ligated DNA was cut into desired length fragments, purified by biotin-streptavidin-mediated pull-down after blunt-end repaired and A-tailed.

The short-read sequencing was conducted on the Illumina NovaSeq 6000. In parallel, a long-read sequencing library with an insert size of 20 kb was constructed and sequenced using the PacBio Sequel II system, which generated high-fidelity (HiFi) reads. All procedures pertaining to library construction and sequencing were outsourced to Berry Genomics (Beijing, China). In total, the sequencing effort yielded 140.74 Gb of data, including 41.61 Gb of PacBio HiFi reads, 28.03 Gb of Illumina short reads, 60.92 Gb of Hi-C derived data, and 10.18 Gb of transcriptomic reads (Table 1). The PacBio HiFi dataset reached a scaffold N50 length of 17.80 kb, while the average read length was 17.45 kb.

**Table 1:** Statistics of the sequencing data used for genome assembly.

Libraries	Insert sizes (bp)	Clean data (Gb)	Sequencing coverage (x)
Illumina	350	28.03	44.60
PacBio HiFi	20 Kb	41.61	66.21
Hi-C	350	60.92	96.91
RNA	350	10.18	—

### Assembling of genomes

The quality of the raw Illumina data was evaluated using BBTools v38.82^15^. The quality control process entailed the removal of duplicate reads through the implementation of the ‘clumpify.sh’ script. Furthermore, for trimming high-quality reads, the ‘bbduk.sh’ script was utilized. Sites exhibiting base quality scores over 20 were selected, and threw out any sequence shorter than 15 bp, eliminated long poly-A/G/C tails in the reads and improved base quality by combining overlapping reads.

The initial genome assembly of *S. princeps* was executed utilizing PacBio HiFi long-read sequencing data through Hifiasm v0.16.1^16^, employing the ‘-l 2’ parameter to adopt a more stringent strategy for filtering out redundant, heterozygous sequences. To further refine assembly accuracy, heterozygous regions were identified and removed by aligning the sequencing reads, produced by Purge_Dups v1.2.5^17^ in conjunction with Minimap2 v2.4^18^, against the assembled genome. Subsequent to this filtration step, mapping of the Hi-C sequencing reads and integrity of the assembly was assessed by the assembled contigs using Juicer v1.6.2^19^. Chromosomal scaffolding was achieved with 3D-DNA v180922^20^, anchoring the primary contigs to specific chromosomes. An in-depth manual curation of the scaffolding results was then conducted using Juicebox v1.11.08^19^ to correct assembly errors and improve overall structural accuracy. Potential contamination within the assembly was systematically evaluated via nucleotide similarity searches using MMseq2 v13^21^, comparing the sequences against the nucleotide from the NCBI and information retrieved from UniVec database. To distinguish between autosomes and sex chromosomes, the finalized assembly was re-aligned with the original PacBio HiFi reads using MiniMap2 v2.17^18^ and chromosomal coverage was quantified by normalizing the raw read depth to chromosome length via SAMtools v1.9^22^.

**Fig. 1:**
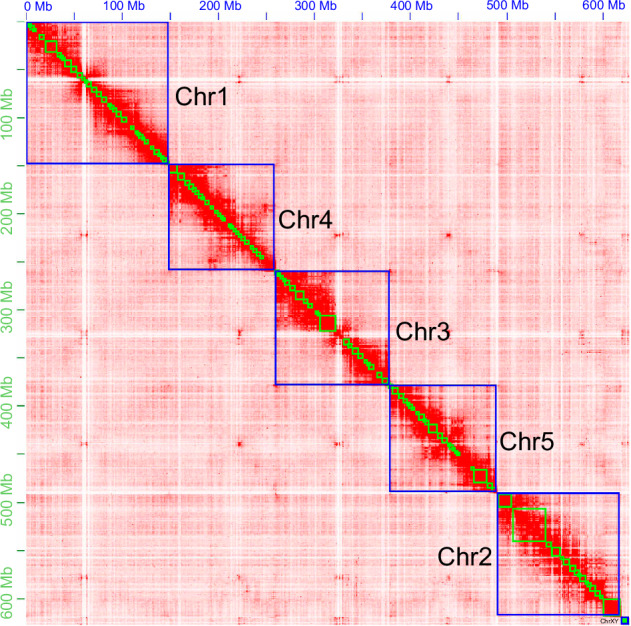
Genome-wide chromosomal heatmap of *Sarcophaga princeps*, with each chromosome framed in blue.

**Fig. 2:**
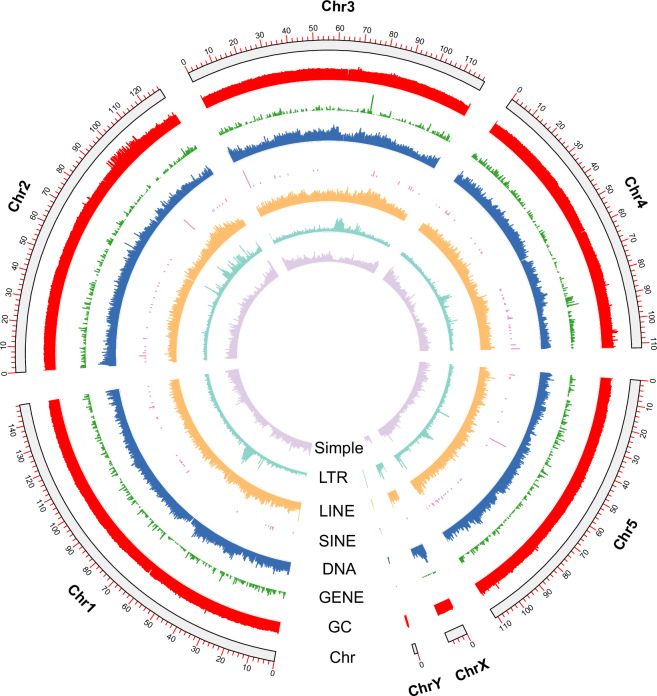
Genome characteristics of *Sarcophaga princeps*. From the outer ring to the inner ring are the distributions of chromosome length, GC content, gene density, transposable elements: DNA transposon, short interspersed nuclear elements (SINE), long interspersed nuclear element (LINE), and long terminal repeats (LTR), and simple repeats.

The finalized *S. princeps* genome assembly reached a chromosome-level resolution, totaling approximately 628.56 Mb in size, composed of 12 scaffolds and 377 contigs, with a GC content of 33.74% (Table 2). It was found that scaffold N50 is 118.89 Mb and contig N50 is 3.64 Mb. Notably, 99.87% of the total contig length, amounting to 627.74 Mb, was successfully anchored to seven chromosomes, with individual chromosome lengths ranging from 1.31 Mb to 148.26 Mb (Table 3; Figs. 1, 2). Chromosomes 6 and 7 exhibited significantly reduced HiFi read coverage (17.28 × and 16.84 × , respectively), approximately half that observed for the remaining chromosomes (Table 3), leading to their designation as the X and Y chromosomes. Additionally, Hi-C interaction maps revealed markedly weaker contact intensities for these chromosomes, further substantiating their classification as sex chromosomes (Fig. 1).

**Table 2:** Genome assembly statistics for *Sarcophaga princeps*.

Assembly	Total length (Mb)	Number scaffolds/contigs (chromosomes)	Scaffold/contig N50 length (Mb)	GC (%)	BUSCO (n = 1,367) (%)			
					C	D	F	M
Hifiasm	2730.50	7791/7791	0.80/0.80	33.79	99.7	98.2	0.1	0.2
Purge_Dups	643.38	569/572	3.59/3.59	33.78	99.1	5.9	0.1	0.8
3D-DNA	643.44	14/572 (7)	118.89/3.59	33.78	99.1	4.7	0.1	0.8
Final	628.56	12/377 (7)	118.89/3.64	33.74	99.1	4.7	0.1	0.8

C: complete BUSCOs; D: complete and duplicated BUSCOs; F: fragmented BUSCOs; M: missing BUSCOs.

**Table 3:** The chromosome length and PacBio HiFi sequencing coverage of *Sarcophaga princeps*.

Chr ID	Chr length (bp)	HiFi data sequencing coverage (x)
Chr1	148,256,136	27.33
Chr2	128,514,504	25.70
Chr3	118,888,638	28.37
Chr4	111,902,932	27.27
Chr5	111,048,456	29.40
Chr6 (ChrX)	7,821,773	17.28
Chr7 (ChrY)	1,312,083	16.84

### Annotation of genomes

The repetitive sequences within the *S. princeps* genome were identified using the LTRStruct pipeline integrated into RepeatModeler v2.0.4^23^, which facilitated the *de novo* construction of a repeat library. To enhance the comprehensiveness of the repeat annotation, this custom library was supplemented with sequences from the Dfam 3.5^24^ and RepBase-20230909^25^ databases. Subsequent genome-wide repeat annotation was conducted using RepeatMasker v4.1.4^26^ employing the consolidated repeat database. The results showed that about 52.99% of the *S. princeps* genome consists of repeated elements. These include 22.92% of elements that could not be classified into known categories, as well as LINE transposons (12.08%), DNA transposons (11.47%), LTR retrotransposons (3.35%), and simple sequence repeats (2.19%), as summarized in Table 4.

**Table 4:** Genome assembly and annotation statistics of *Sarcophaga princeps*.

Content	Sarcophaga princeps
**Genome assembly**	
Assembly size (bp)	628,555,410
Number of pseudo-chromosomes (sizes)	7 (627,744,522 bp)
Number of scaffolds/contigs	12/377
Longest scaffold/contig (Mb)	148.26/34.40
N50 scaffold/contig length (Mb)	118.89/3.64
GC content (%)	33.74
BUSCO completeness (%)	99.1
**Protein-coding genes**	
Number	14,348
Mean gene length (bp)	12,899.0
Number of exons per gene	4.8
Mean exon length (bp)	437.4
Number of CDSs per gene	4.6
Mean CDS length (bp)	376.8
Number of introns per gene	3.7
Mean intron length (bp)	3,027.0
BUSCO completeness	98.8%
**Repetitive elements**	
Size (Mb)	333.04 (52.99%)
DNA transposons (Mb)	72.22 (11.47%)
SINEs (kb)	54.14 ( < 0.001%)
LINEs (Mb)	76.01 (12.08%)
LTRs (Mb)	21.09 (3.35%)
Unclassified (Mb)	144.09 (22.92%)
Simple repeat	13.79 (2.19%)
**ncRNA**	
Number of ncRNA	3084
rRNA	560
miRNA	77
snRNA	150
tRNA	756
lncRNA	2

For non-coding RNA (ncRNA) annotation, two complementary approaches were employed. Firstly, Infernal v1.1.4^27^ was used to identify rRNAs, miRNAs, snRNAs, and other ncRNA species by aligning the genome sequences against the Rfam v14.10 database^28^. Secondly, tRNAscan-SE 2.0.9^29^, was applied to predict the presence of tRNA genes. These analyses collectively identified 3,084 ncRNAs in the genome of *S. princeps*, comprising 560 rRNAs, 77 miRNAs, 150 snRNAs, 756 tRNAs, 2 ribozymes, and 2 long non-coding RNAs (lncRNAs), as detailed in Table 4.

The annotation of protein-coding genes involved a combination of transcriptome-based evidence, *ab initio* gene prediction, and homology-based methods, orchestrated via the MAKER v3.01.03 pipeline^30^. Transcript alignment was performed using HISAT2 v2.2.1^31^. and subsequent genome-guided transcriptome assembly was conducted with StringTie v2.1.6^32^. For *ab initio* predictions, BRAKER v2.1.6^33^ was utilized, which incorporates GeneMark-ES/ET/EP 4.68_lic^34^ and Augustus v3.4.0^35^ both of which were automatically trained using RNA-seq alignments and protein homologs sourced from OrthoDB v11^36^. Homology-based prediction was conducted using GeMoMa v1.9^37^ with the parameters “GeMoMa.c = 0.4” and “GeMoMa.p = 10”, relying on conserved intron positions and protein sequences from five reference species: *Anopheles gambiae* (GCF_943734735.2)^38^, *Aedes aegypti* (GCF_002204515.2)^39^, *Sarcophaga bullata* (GCA_005959815.1)^40^, *Drosophila melanogaster* (GCF_000001215.4)^41^ and *Lucilia cuprina* (GCA_022045245.1)^42^. Finally, the annotation process found 14,348 protein-coding genes in the *S. princeps* genome. On average, each gene spanned approximately 12,899.0 bp pairs and was composed of 4.8 exons, 3.7 introns, and 4.6 coding sequences (CDS). The average lengths of exons, introns, and CDS were calculated to be 437.4 bp, 3,027.0 bp, and 376.8 bp, respectively (Table 4). The completeness of the protein sequences was assessed using BUSCO, resulting in a high score of 98.8% (n = 1,367). This encompassed 85.0% single-copy, 13.8% duplicated, 0.1% fragmented, and 1.1% missing BUSCOs, indicating high-quality predictions.

To functionally annotate genes, we conducted comprehensive searches against the UniProtKB database employing the sensitive mode of Diamond v2.0.11.1^43^ using the parameters “–very-sensitive -e 1e-5” to ensure high-accuracy matches. Additionally, we utilized the annotation tools eggNOG-mapper v2.1.5^44^ and InterProScan 5.53-87.0^45^ to assign Gene Ontology (GO) terms, identify metabolic and signaling pathways through KEGG and Reactome databases, and detect protein domains. The InterProScan analyses incorporated data from Pfam^46^, SMART^47^, Superfamily^48^, and CDD^49^, among four other integral domain databases, to ensure broad domain coverage. The data produced by these tools was brought together to create a single functional prediction dataset. As a consequence, 13,826 genes which represent 96.36% of the *S. princeps* genome, were included in the UniProtKB database. We also learned that 11,730 structural domains in proteins from InterProScan were associated with protein-coding genes. Combined annotations indicated that the *S. princeps* genome comprises 12,191 entries within the Clusters of Orthologous Groups (COG) categories, 8,109 KEGG orthology terms, 7,202 GO annotations, 5,002 KEGG pathways, and 2,705 enzyme classification codes (Table 5), as derived through the integration of eggNOG and InterProScan outputs. Furthermore, we visualized genomic features including repeat element distribution, gene density, and GC content across individual pseudochromosomes using^50^ (Fig. 2).

**Table 5:** Function annotation statistics of *Sarcophaga princeps*.

Function annotation	*Sarcophaga princeps*
Number of genes matching Uniprot records	13,826
Number of genes labeled as “Uncharacterized protein”	439
Number of genes labeled as “unknown function”	531
Number of genes with InterProScan annotations	11,730
Number of genes with GO items from InterProScan annotations	7,202
Number of genes with eggNOG annotations	13,468
Number of genes with GO items from eggNOG annotations	10,122
Number of genes with Enzyme Codes (EC) from eggNOG annotations	2,705
Number of genes with KEGG ko terms from eggNOG annotations	8,109
Number of genes with KEGG pathway terms from eggNOG annotations	5,002
Number of genes with COG Functional Categories from eggNOG annotations	12,191
Number of genes with GO items (combining InterProScan and eggNOG results)	11,052
Number of genes with KEGG pathways items (combining InterProScan and eggNOG results)	5,002

## Data Records

The complete dataset, including raw sequencing reads and the assembled genome of *S. princeps*, has been submitted to the National Center for Biotechnology Information (NCBI). Hi-C, transcriptomic, Illumina, and PacBio HiFi data are accessible via their respective accession numbers SRR30518403^51^, SRR30518404^52^, SRR30518405^53^, and SRR30518406^54^. The finalized genome assembly is available in the NCBI Assembly database under a designated accession number GCA_049996135.1^55^. Supplementary data pertaining to repeats, gene annotations, and functional predictions are publicly available on Figshare^56^.

## Technical Validation

To evaluate the assembly’s completeness and integrity, we implemented two complementary validation approaches. First, Benchmarking Universal Single-Copy Orthologs BUSCO v5.0.4^57^ was employed, using the Insecta reference gene dataset (n = 1,367). The assembled genome demonstrated exceptional completeness, achieving a BUSCO score of 99.1%, with 94.4% of genes identified as single-copy orthologs, 4.7% as duplicated, only 0.1% fragmented, and 0.8% missing. Secondly, alignment-based validation was performed using Minimap2 in conjunction with SAMtools. The alignment of sequencing reads to the assembled genome demonstrated high mapping rates: 99.80% for PacBio reads, 94.06% for Illumina reads, and 77.00% for RNA-seq reads. These metrics collectively underscore the high fidelity and robustness of the genome assembly.

## Data Availability

No specific scripts were developed for this project. All data processing and bioinformatics analyses were conducted using publicly available software, following protocols and manuals provided by each respective tool.
